# National Dental Association, Omaha, 1898

**Published:** 1898-10

**Authors:** 


### PROCEEDINGS.

### National Dental Association, Omaha, 1898.

The National Dental Association held its first annual meeting in Omaha, Nebraska. The meetings were held in the Creighton Medical College Building, which afforded ample facilities for all departments of the work. The meeting was called to order on the morning of August 30th, with the President, Dr. Thomas Fillebrown, occupying the chair. After an opening prayer by Dr. J. Taft, the Mayor of the city, Hon. Frank N. Moore, delivered a witty address of welcome, to which Prof. James Truman made an eloquent response.

After the usual routine business, reports of officers and committees, and the transaction of some miscellaneous business, the President read his Annual Address, of which we give a brief abstract: He spoke of the influence of organization, as essential to the success of any movement in which the combined effort of individuals is desired. Nations, peoples and communities require formulated rules for their government and control. Laws are the measure of progress; good laws are not found in corrupt ages, while an enlightened people will not tolerate evil laws. Applying the principle of law to our organization he said that in the past it was as good as the material of which it was composed would afford. The present Association will be no better than the knowledge, culture and efforts of its members make it. The things most essential to the success of an organization are brains, education, enthusiasm and an unselfish devotion, that will bring to, and lay upon the altar of science, the sweet incense of the best efforts of every member. Professional relations must be governed by an entirely unselfish principle. He then spoke of the two distinctly new features of the present Constitution; first, the creation of the divisions of the East, the West and the South; a feature of cosmopolitan character, which must awaken wider interest in the meetings and increase the membership, while guarding against sectional control. The second distinctively new feature is the formation of branches, through the influence of which it is expected that many will become actively connected with the National Association, working in harmony with it, who would not otherwise join its ranks. Its practical and successful working has been shown by the Southern Branch during the present year, and repetition of such success may be looked for. With branches working in harmony with the parent body, the proceedings published in the volume of the National Association will furnish a complete record of the scientific progress of the profession during the year. The president spoke of the manner of securing delegates as being perhaps too complicated, the limitation of delegates to State Societies perhaps keeping many good men out of the Association. The true value of professional organization lies in the wider diffusion of knowledge, culture and refinement; hence, open wide the doors and go out into the streets and byways and compel men to come to the feast. Honor is accorded to those who impart their knowledge the most freely. He spoke of the future of the Association as appearing especially bright and encouraging. Whatever of shadow may have hung over the profession in the past, whatever of prejudice, ambition or adverse interests may still exist, there is light upon its pathway in the future. The future promises much for the increase of scientific knowledge, the advancement of professional interests, and the promotion of the welfare of humanity.

At the conclusion of the president's address, upon motion a commitee was appointed to take into consideration the suggestions and recommendations contained in it and report upon the same at the subsequent session of this body.

The committee consisted of Dr. H. A. Smith.

ABSTRACT OF PAPER READ BY DR. B. HOLLY SMITH, ENTITLED "REMOVAL OF THE DENTAL PULP."

In proportion as medicine has grown scientific and skillful, less dependence is placed on the therapeutic effects of drugs for the alleviation of human ills. Secondary effect has assumed greater importance, and possible evils attending administrations have outweighed the advantage of immediate relief.

For more than half a century practitioners of dentistry have relied upon the application of arsenious acid to the dental pulp, with a view to its destruction. Has any one found the exact limits of the destructive power of that agent? Has the use of this material been attended with no secondary evils? That pericemental disturbances do frequently occur, can be stated without fear of dispute; that arsenic may be the cause has long been the thought of the writer, an opinion formed through observation of the contrast between teeth made pulpless through surgical procedure, pure and simple, and those from which the pulps have been removed after the application of arsenical preparations. The well-known germicidal qualities of arsenic would indicate that no trouble should be expected from the contents of the tubuli which have been subject to arsenical application, and sealed from the chance of subsequent infection; yet trouble not infrequently does arise in after years, even where there is reasonable certainty that the apex was entirely closed.

Why? Is it possible that a sleeping volcano has been located in the apical space, by the application of arsenic; that the effect of the agent has not been limited to the confines of the pulp canal and dentinal tubuli; that the area of tissue affected breaks down, in seasons of depressed vitality, or becomes infected through the medium of the circulation? The apical opening may be infinitesimal, but it must be large in comparison with the tubuli in the dentine. Why may not the nerves and vessels in the apical space and a considerable area of the pericemental membrane have a share in the action of the arsenic? There is no positive evidence of a well-defined line of demarcation in the effect, even after the agent passes the point where it has power to destroy. May it not deplete and incapacitate the tissues until danger is invited and may subsequently follow?

Dr. Smith then quoted from Dr. J. Foster Flagg (1877) on the effects of arsenic taken into the bloodways; from Arkoog, on the inflammation concomitant with the dying of the pulp; from Dr. Burchard's recently published work, "Dental Pathology and Therapeutics," on the specific action of arsenic; from Dr. I. P. Wilson (1888) on its effects upon the cementum, and from Dr. Kirk (1898) on inflammatory action and bacterial invasion, thence deducing an explanation of the pathological expressions occurring in the apical space of teeth which have been treated with arsenic, drawing the conclusion that arsenic should no longer enjoy its universal popularity, but should give place to surgical procedure.

A change in this respect involves not so much the absence of other means as aversion to change of practice, and the well-fixed habits of treating teeth without charge, giving two hours to filling a cavity with gold, and five minutes to the destruction of a pulp. If, as we claim, we are dental surgeons—not mere cavity-stoppers—this is not right, and must be changed.

The conditions essential for pulp removal by operative procedure were then considered, and the method of treatment outlined as follows: To the dental surgeon, as to the general surgeon, the necessity for operative procedure must be evident, and the field of operation be in as favorable condition as can be obtained. No effort should be made to remove a pulp in a high state of inflammation; employ antiphlogistic agents until irritation has subsided. Flood the cavity with a tepid solution of bicarb. soda. Then close cavity with a pledget of cotton saturated in cocaine hydrochlorate for a few moments, avoiding pressure, and taking great care not to cause pain. Adjust a concave disk of vacuum-cavity material, so that its edges will impinge upon the walls of the cavity and prevent pressure. Under this disk place a pledget of cotton saturated with oil of cloves. Cover with temporary stopping, paint the gums with aconite and iodine, equal parts, and dismiss patient until one, or preferably two hours, can be given to the operation of removal, when, if conditions are favorable, the pulp may be cocainized with the electric current and removed, using minimum amount of current, and a saturated solution of cocaine. If cataphoresis is not successful in the highest sense, supplant by a general anesthetic, preferably nitrous oxide.

When teeth containing living pulps are to be excised or ground down, advantage can be taken of the opportunity to remove the pulp under the influence of the shock of excision, which can be done with no pain. Cut the enamel with disk or bur, leaving for the forceps only so much as will not require stress or violence. Having ready in the engine a cone-shaped bur, clean and sharp broach, etc., remove the pulp immediately by passing the broach quickly to the apex, twisting the pulp out in toto.

The most inviting field for the surgical operation is found in single root teeth, but it can be employed in the molars if the effort is painstaking. Surgery is not only more scientific, but is also proven to be more successful. A plea for this change in method should, therefore, not pass unheeded.

DISCUSSION.

In the discussion of this paper, DR. BARRETT said there was much in the subject which needs clearing up. The author of the paper attributes the death of the pulp, under arsenical applications, to the action of the arsenic on nerve tissue, but arsenic is a corrosive agent; it induces necrosed conditions in osseous tissue. If death of the pulp is due to constriction at the apical foramen, from forced stasis, how is it caused when there is no open foramen? If the arsenic is not securely sealed in the tooth-cavity, the effects are just as fatal upon the buccal tissues as upon the pulp tissue itself.

DR. HUNGERFORD thought the spirit of the paper was not an exemplification of pathological conditions, the use of therapeutic agents and surgery being the keynote. He is opposed to the contamination of the blood stream by the introduction of therapeutic agents by hypodermic injections. He treats pulps surgically, varying the method according to conditions. After the application of arsenic to the pulp, death is due to congestion in the pulp chamber, not at the apical foramen.

DR. PATTERSON: Pericemental inflammation, after pulp removal, can always be avoided by removing the pulp by surgical methods. Cocaine can be forced into the pulp until it is completely anesthetized, using very finely pulverized crystals of cocaine. With the chlorid of ethyl spray the pulp can be so congealed that cocaine can be forced into it by means of pressure with spunk, cotton or unvulcanized gutta-percha. The pulp can then be removed absolutely without pain.

Dr. Taft abandoned the use of arsenic twenty years ago. Its effects are objectionable, not only upon the pulp itself but by extending to other tissues. The results are too uncertain. With the increase of facilities, other methods are so efficient that there is no longer any excuse for its use. Cocaine, finely pulverized and dissolved, is readily taken into the pulp by absorption, until it is so anesthetized that it can be removed with as little pain as a paring from the finger nail. When the pulp is removed by surgical procedure and all soft tissues removed from the canal and hemorrhage arrested, then is the time to close it. Seal it up immediately, and there is no liability of any changes pernicious to its welfare.

Dr. Watkins prefers, when he has a large cavity with free access, to punch it out with a slim piece of orange wood. It is instantaneous, the pulp is removed entire, and the tooth is ready for filling.

Dr. Barrett: Those who oppose arsenic as a dangerous drug, advise the use of cocaine! Which is the most dangerous? It is "jumping from the frying-pan into the fire."

Dr. B. H. Smith: The surgeon who uses cocaine knows the liabilities. He is prepared to combat evil effects, which he knows how to counteract. The trouble with arsenic is, that we do not know how far-reaching its effects may be in any case.

Dr. Crawford spoke of the importance of preventing septic infection (which he prefers to call toxic invasion) by stopping each root as soon as the canal is emptied, not leaving it open to serve as a means of self-destruction, while operating in the other canals.

Dr. Smith, in closing the discussion, said that he had desired to present the record of some clinical cases, but had decided to wait and study them further.

ABSTRACT OF PAPER READ BY DR. L. E. CUSTER, ENTITLED "THE IMPROVED ELECTRIC OVEN AND THE MEANS OF DETERMINING THE HEAT IN FUSING PORCELAIN."

In the improved oven the perfection of detail has all been in the direction of producing the highest heat in the oven cavity, with the lowest possible heat of the platinum wire, which, in the

Custer oven, has been reduced step by step, by perfecting every available point that might be of advantage until it is reduced, so that it will now stand hundreds and perhaps thousands of heatings. Dr. Thomas reports having used nearly one hundred ounces of close material without a burnout, a conservative estimate of which would be that the oven was heated from 800 to 1,000 times. This result has been attained by making every inch of the walls heat-producing surfaces, (except the two small openings) arranging wires over the entire surface as closely together as can be without touching laterally, the wires being also brought to the surface of the clay, placing them only deep enough to be caught, so that nothing intervenes between the wire and the object heated. The wires are also placed closer together as the distance from the center increases, so that all portions of the cavity are equally heated. This idea, original with Dr. Custer, is now being copied in electric culinary utensils. The discovery of the fact that the negative end of the wire becomes about one-fifth hotter in a constant electric current than the positive, led to the method of winding the oven with the negative end of the wire larger than the positive end and solves the most perplexing problem, and the oven may now be said to be perfect in every detail.

Dr. Custer described two methods of determining the heat of the oven. One is by the use of the thermometer which he inserts into a clay cup outside of the oven, having a twenty-gauge platinum wire, of which one end enters the oven cavity, the other terminating in the cup, which encloses the bulb of the thermometer, the heat of the cup end being always exactly proportionate to the end of the wire in the oven.

Another and better method, one under complete control, is by the use of an arc light. By means of a little frame, placed on the top of the oven, a small carbon is held opposite the upper opening; another carbon, which is movable, being held in a guide. When it is desired to see the condition of the porcelain, the movable carbon is pushed toward the fixed carbon until the two are in contact. The movable carbon is then withdrawn a short distance and a brilliant arc will be struck, throwing such a strong light into the oven that the operator sees the porcelain melt as distinctly as if in the open air. There is no guess-work, and the eye is relieved of the great strain of trying to see an indistinct object.

DISCUSSION.

In the discussion of Dr. Custer's paper, Dr. H. A. Smith asked if any method had been devised by which platinum scraps can be melted by means of the electric current, leaving the platinum equal to the original?

Dr. Custer replied that when platinum is fused in the presence of carbon, so much carbon is taken up that the result may be compared to the conversion of iron into steel, giving a metal having the qualities of platino-iridium. If, however, it is desired that the platinum shall retain its softness it is only necessary that it be melted in the presence of lime, when it will be as soft and ductile as the new platinum which you buy.

Dr. Tileston inquired whether the arc light illumination of the oven by Dr. Custer's method is superior to that employed for the Downie furnace?

Dr. Custer replied that it differed as much as the arc light differs from an incandescent light.

Dr. Molyneaux spoke of the great importance of being able to recognize, with exactness, the fusing point of porcelain. As we acquire more accurate methods, and less guess-work, more porcelain work will be done, and better results will be obtained.

---

The widow of the late Sir Morell Mackenzie is earning her living as a modiste, and is about to sell her late husband's library to raise money to live on. What a commentary on the ups and downs of this life, but more especially upon the unprofitableness of medical practice.—*Western Med. Review.*

---

### Pride Before A Fall.

Pedagogic father is trying to show off his supposed precocious boy before company.—"My son, who would you rather be, Shakespeare or Dewey?" Little son meditates awhile and says, "I'd rather be Dewey." "What, my son; and why?" "'Cause he ain't dead."—*New York Weekly.*

